# Atomic Interactions and Order–Disorder Transition in FCC-Type FeCoNiAl_1−*x*_Ti_*x*_ High-Entropy Alloys

**DOI:** 10.3390/ma15113992

**Published:** 2022-06-03

**Authors:** Ying Wu, Zhou Li, Hui Feng, Shuang He

**Affiliations:** 1 College of Mechanical and Vehicle Engineering, Hunan University, Changsha 410082, China; 2 College of Artificial Intelligence and Big Data for Medical Sciences, Shandong First Medical University & Shandong Academy of Medical Sciences, Jinan 250117, China; lizhou_alfred2011@hotmail.com; 3 State Key Laboratory of Advanced Design and Manufacturing for Vehicle Body, Hunan University, Changsha 410082, China; fenghuiff@hnu.edu.cn; 4 School of Materials Science and Engineering, Xiangtan University, Xiangtan 411105, China; shuanghe@xtu.edu.cn

**Keywords:** high-entropy alloy, first-principles calculation, thermodynamic simulation, atomic interaction, order–disorder transition

## Abstract

Single-phase high-entropy alloys with compositionally disordered elemental arrangements have excellent strength, but show a serious embrittlement effect with increasing strength. Precipitation-hardened high-entropy alloys, such as those strengthened by L1_2_-type ordered intermetallics, possess a superior synergy of strength and ductility. In this work, we employ first-principles calculations and thermodynamic simulations to explore the atomic interactions and order–disorder transitions in FeCoNiAl_1−*x*_Ti_*x*_ high-entropy alloys. Our calculated results indicate that the atomic interactions depend on the atomic size of the alloy components. The thermodynamic stability behaviors of L1_2_ binary intermetallics are quite diverse, while their atomic arrangements are short-range in FeCoNiAl_1−*x*_Ti_*x*_ high-entropy alloys. Moreover, the order–disorder transition temperatures decrease with increasing Ti content in FeCoNiAl_1−*x*_Ti_*x*_ high-entropy alloys, the characteristics of order–disorder transition from first-principles calculations are in line with experimental observations and CALPHAD simulations. The results of this work provide a technique strategy for proper control of the order–disorder transitions that can be used for further optimizing the microstructure characteristics as well as the mechanical properties of FeCoNiAl_1−*x*_Ti_*x*_high-entropy alloys.

## 1. Introduction

High-entropy alloys (HEAs) are usually defined as those alloys having at least five major metallic elements and atomic concentrations in the range between 5 and 35 at.%. The premise on which these alloys have been conceptualized is high configurational entropy, due to a possible random mixing of constituent alloying elements in these multi-principal-element alloy systems, which reaches a maximum when the alloy is equiatomic [1,2,3,4]. Based on the entropy concept, the so-called HEAs are also generally defined as alloys having a high configurational entropy of more than 1.5 *R* (*R* being the gas constant) [5]. Due to the excellent strength and ductility of the single-phase HEAs at cryogenic temperatures, one of the main focus areas of HEAs studied earlier was to design a single-phase solid solution phase by adjusting the configurational entropy, however, they usually possess low ductility when the high strength is achieved, which makes them inappropriate for engineering applications. Therefore, extensive efforts have been turned toward dual-phase or multi-phase HEAs for achieving a combination of strength and ductility [6,7,8,9].

In recent work, the mechanical properties of HEAs containing ordered L1_2_ precipitate have been investigated comprehensively. It was supposed that the formation of precipitate phases could contribute to achieving a balance between ductility and strength [10,11,12,13,14,15]. Lu et al. found that the balanced tensile properties were achieved in an fcc FeCoNiCr HEA due to the formation of a unique microstructure of the L1_2_–Ni_3_(Ti,Al) nano-sized coherent reinforcing phase in the matrix [10]. The same was true in the investigation by Kai et al. for CoCrNi medium-entropy alloy containing nanoscale L1_2_-(Ni, Co, Cr)_3_(Ti, Al)-type particles, compared to the single-phase CoCrNi medium-entropy alloy, the yield strength and the tensile strength of the precipitation-strengthened CoCrNi medium-entropy alloy were increased by ~70% to ~750 MPa and ~44% to ~1.3 GPa, respectively, whereas a good ductility, ~45%, was still achieved [11]. In an experimental study of the effect of coherent L1_2_ nano-precipitates on the tensile behavior of a novel-designed Al_0.2_CrFeCoNi_2_Cu_0.2_ [12], Wang et al. found that the presence of L1_2_ nano-precipitates resulted in increases of 259 MPa and 316 MPa in the yield strength and ultimate tensile strength, respectively, accompanied by the maintenance of a high elongation (30.4%). Moreover, it was reported by Gwalani et al. that the L1_2_ phase could be stabilized by Cu addition in the Al_0.3_CrCuFeNi_2_ HEA [13]. A similar mechanical response was also observed when modulating the Al and Ti contents in the FeCoNiAl_*x*_Ti_*y*_ HEAs [16,17], while the Ti/Al ratios ranging from 0.7 to 2 would prefer the formation of the L1_2_ phase in the CoCrFeNi-based HEAs [14]. This was also confirmed by Joseph et al. in the Al–Co–Cr–Fe–Ni–Ti HESAs, where the authors found that the phase equilibrium and yield strength of the Al–Co–Cr–Fe–Ni–Ti HEAs could be affected by the (Al/Ti)-ratio [18]. Moreover, the chemical instability of the supersaturation and migration of grain boundaries could cause the formation of cellular precipitate, which in turn affects the tensile properties of the precipitation-strengthened HEAs [15].

The fundamental understanding of the thermodynamic and mechanical stability of precipitates in HEAs is of major importance for the design of new alloys and for an accurate thermodynamic description of the materials containing such precipitates using atomistic modeling and thermodynamic simulation, i.e., first-principles calculations and the calculation of phase diagrams (CALPHAD) method [19]. Using first-principles calculations, Widom et al. found that the configurational entropy could stabilize a single body-centered cubic (BCC) phase from 1700 K up to melting, while a complex intermetallic was favored at lower temperatures [20]. Hu et al. reported that the HfNbTaTiZr HEA decomposed most favorably to BCC NbTa-rich and HfZr-rich phases below the critical temperature of 1298 K [21]. Lai et al. calculated the formation enthalpy and cohesive energy of a list of potential inter-metallic compounds in the FeTiCoNiVCrMnCuAl HEA and showed that FeTi, Fe_2_Ti, AlCrFe_2_, Co_2_Ti, AlMn_2_V and Mn_2_Ti phases were preferential in the formation process of the FeTiCoNiVCrMnCuAl HEA [22]. Using density functional theory (DFT) calculations, the ordering-induced elastic anomaly in the FeCoNiAl_1−*x*_Ti_*x*_ were investigated, it was found that the Ti addition could be attributed to the presence of an ordered L1_2_ phase [23]. Sluiter et al. investigated the order–disorder transition in NbMoTaW HEA, they found that the B2(Mo;Ta) and B32(Nb;W) were stable from an energetic point of view in the ground state and revealed a B2(Mo,W;Ta,Nb) ordering at ambient temperatures [24]. Using CALPHAD methods, the content of L1_2_–Ni_3_(Ti, Al) phase contents in NiCoAlTiFeCr HEA [25], the grain size as well as the volume fraction of L1_2_ precipitates in FeCoNiAlTi HEA [26], and the phase stability of L1_2_-(Ni,Co,Cr)_3_(Ti,Al) in CoCrNi-based medium-entropy alloys were investigated [11]. The results indicated that the CALPHAD method could be used as a practical tool for designing microstructure and mechanical properties of HEAs.

It is known that the phase transformation in HEAs involves the movement of atoms through the metals to rearrange themselves to form the new phases or precipitates, which is also linked to solute mobility and interactions at finite temperatures. Unfortunately, little is known about the atomic interactions in HEAs, even that the atomic interactions of components in HEAs play a critical role in the order–disorder transition in the alloys [24], however, no complete phase diagram is currently available to directly assist in designing FeCoNiAlTi HEAs with desirable micro- and nano-structures. Moreover, it is already known that the Ti addition enhanced the mechanical strengthening of FeCoNiAl_1−*x*_Ti_*x*_ HEAs [23], but the phase stability and order–disorder transition was not clear in this case. Therefore, the effect of Ti content on the phase stability and order–disorder transition needs to be investigated further. Such theoretical investigations can be used as a strategy for designing the chemical composition and microstructure of FeCoNiAl_1−*x*_Ti_*x*_ HEAs.

In this work, the phase stability of FeCoNiAl_1−*x*_Ti_*x*_ HEA was investigated based on the consideration of the interatomic interactions, atomic ordering, and phase transformation. Specifically, the pair interactions of component species in an fcc single-phase lattice were investigated to reveal how these elements interact with each other and how the interactions contribute to the chemical ordering and phase transformation of the FeCoNiAl_1−*x*_Ti_*x*_ HEAs. Furthermore, Monte Carlo statistical thermodynamic simulations of elevated temperature ordering in FeCoNiAl_1−*x*_Ti_*x*_ HEAs were conducted and compared with the solidification process simulation using the CALPHAD method. Such an analysis can be directly linked to the precipitation hardening phenomena and used in a consistent approach to identify the influence of each element as well as precipitates in the thermodynamic and mechanical properties of FeCoNiAl_1−*x*_Ti_*x*_ HEAs, the results of this work will provide solutions for composition and structure optimization to improve the mechanical properties of the FeCoNiAl_1−*x*_Ti_*x*_ HEAs.

## 2. Computational Method

### 2.1. Atomic Interactions

In this study, considering atomic interactions in face-centered cubic (fcc) Ni matrix [27], we obtain the atomic interactions from a set of the total energies of supercells where positions in a given cluster are occupied by atoms in different configurations. These were calculated from two representative cases:

(i) In the binary system of Ni–X, the X–X solute pair interaction at the *p*th coordination shell is calculated by [27]:
  (1) $$\begin{array}{c} V_{p}=E_{p}^{\mathrm{XX}}+E_{\mathrm{Ni}}-2E_{\mathrm{imp}}^{X}, \end{array}$$
  where $E_{p}^{\mathrm{XX}}$ is the total energy of a supercell with two X atoms at the *p*th coordination shell, $E_{\mathrm{imp}}^{X}$ is the total energy of a supercell with one X atom, and $E_{\mathrm{Ni}}$ is the total energy with no impurity;
(ii) In the ternary system of Ni–X–Y, the X–Y solute pair interaction in the *p*th coordination shell is calculated by:
  (2) $$\begin{array}{c} V_{p}=E_{p}^{\mathrm{XX}}+E_{p}^{\mathrm{YY}}-2E_{p}^{\mathrm{XY}}, \end{array}$$
  where $E_{p}^{\mathrm{YY}}$ is the total energy of a supercell with two Y atoms at the *p*th coordination shell, and $E_{p}^{\mathrm{XY}}$ is the total energy of the supercell with one X atom and one Y atom at the *p*th coordination shell. In this work, a negative interaction energy indicates the attraction between solutes.

Although the size mismatches of atoms in FeCoNiAl_1−*x*_Ti_*x*_ HEAs are rather moderate, due to the equal or nearly equal atomic ratio in HEAs, local lattice relaxations play a critical role in alloy energetics [28,29]. Therefore, the differences between chemical and total interactions are calculated and defined as the strain-induced (SI) interaction:

(3) $$\begin{array}{c} V_{p}^{\mathrm{SI}}=V_{p}^{\mathrm{total}}-V_{p}^{\mathrm{chemical}}, \end{array}$$

where $V_{p}^{\mathrm{total}}$ is calculated by relaxing the atoms in the supercells, while for the $V_{p}^{\mathrm{chemical}}$, the atom positions in the supercells are fixed. In both cases, the shape of the supercells is kept at the equilibrium lattice constant [30].

### 2.2. Thermodynamic Monte Carlo Method

In the statistical thermodynamic Monte Carlo (MC) simulations, the atomic interactions obtained from DFT calculations are used to capture the chemical degree of freedom of alloys. The configurational ordering energy of the FeCoNiAl_1−*x*_Ti_*x*_ is mapped onto an Ising Hamiltonian employing atomic interactions [24]:

(4) $$\begin{array}{c} \Delta H_{\mathrm{conf}}=\frac{1}{2}\sum_{\mu \nu }\sum_{p}^{p_{max}}V_{\mu \nu }^{\left(p\right)}\sum_{n,m\in p}\delta c_{\mu }^{\left(n\right)}\delta c_{\nu }^{\left(m\right)}, \end{array}$$

where the second sum runs over all pairs *p*, while $V_{\mu \nu }^{\left(p\right)}$ are the atomic interactions between atomic species μ and ν in FeCoNiAl_1−*x*_Ti_*x*_ HEAs . The first sum runs over the N−1 species μ, ν and $\delta c_{\mu }^{\left(n\right)}$, and $\delta c_{\nu }^{\left(m\right)}$ denotes the concentration variables at lattice sites *n* and *m*. The N−1 independent concentration fluctuations $\delta c_{\mu }^{\left(n\right)}$ are given as ν and $\delta c_{\mu }^{\left(n\right)}$ = $c_{\mu }^{\left(n\right)}-c_{\mu }$ with concentration $c_{\mu }$. The $c_{\mu }^{\left(n\right)}$ is the site-occupation variable which is equal to 1 (0) if the atom at site *i* is (is not) occupied by species μ. In most cases, higher-order terms are found to be quite small and can be neglected [27].

### 2.3. Formation Enthalpy of L1_2_-A_3_B Precipitate

The formation enthalpy (ΔH) of a four-atom L1_2_-A_3_B intermetallic compound is calculated by using the following equation [31,32]:

(5) $$\Delta H\left(A_{3}B\right)=\frac{E_{0}\left(A_{3}B\right)-3E_{0}\left(A\right)-E_{0}\left(B\right)}{4},$$

where $E_{0}\left(A_{3}B\right)$ is the total energy of the L1_2_-A_3_B intermetallic compound, $E_{0}$(A) and $E_{0}$(B) are the zero-Kelvin total energies of the pure elements at their reference states, namely, fcc, bcc, hcp, fcc, and hcp, are taken as the ground state crystal structures for Ni, Fe, Co, Al, and Ti, respectively. The total energies of the L1_2_-A_3_B precipitates, as well as Ni, Fe, and Co, are calculated in the ferromagnetic state.

### 2.4. First-Principles Method

Density functional theory (DFT) total-energy calculations for FeCoNiAl_1−x_Ti_x_ HEAs within DFT were performed using the projector augmented-wave (PAW) method [33] as implemented in the Vienna ab initio simulation package (VASP) [34,35,36]. All the calculations were performed using the Perdew–Burke–Ernzerhof (PBE) form for the generalized gradient approximation (GGA) [37,38]. The atomic interactions are determined in the dilute limit using a 3 × 3 × 3 repetition of the initial fcc 4-atom supercell in the ferromagnetic state of Ni. In order to keep the cubic symmetry of the underlying fcc lattice, which should be preserved on average in real alloys, the translation vectors of supercells are fixed. The integration over the Brillouin zone is performed using the 4 × 4 × 4 Monkhorst-Pack grid [39] for the supercell. In the calculations for the atomic interactions for FeCoNiAl_1−*x*_Ti_*x*_ HEAs, atomic positions are relaxed while the shape of the supercells is fixed. The *k*-point density is equivalent to the 12 × 12 × 12 k-mesh grids for a four-atom fcc unit cell scale, namely, a 4 × 4 × 4 k-mesh density, is used for a 3 × 3 × 3 conventional fcc supercell. It is helpful for avoiding systematic errors [27,32,40].

Other details for all the VASP-PAW calculations are as follows. The convergence criteria are 10^−5^ eV per cell. All the atomic positions inside the supercells are relaxed until the forces acting on the atoms are less than 9 × 10^−3^ eV/Å. The lattice constant $a_{0}$ is chosen as 3.52 Å, being between the derived *T* = 0 K value from experimental [41] and theoretical values [30,42]. Plane waves are included up to 500 eV, which is found to be sufficient for the accurate convergence of total energies. The atomic interactions are calculated using a 3 × 3 × 3 conventional fcc supercell [42,43].

### 2.5. CALPHAD Method

The precipitation temperatures of the L1_2_ phase were calculated using Thermo-Calc 2021b [44] with TCHEA5 databases [45], with the aim of comparing them with those predicted in the Monte Carlo calculations. In the present study, the Sheil–Gulliver model was adopted to simulate the solidification process and the phase precipitation sequence of the FeCoNiAl_1−*x*_Ti_*x*_ HEAs with different chemical compositions. The assumption of the Sheil model is that the speed of diffusion in the liquid phase is rapid enough to achieve an even composition, and only local equilibrium is reached at liquid/solid interfaces. The calculated results for different alloys are shown in Section 3.5.

## 3. Results and Discussion

### 3.1. Atomic Interactions of Solute Pairs in FeCoNiAl_1−x_Ti_x_ HEAs

**Figure 1:**
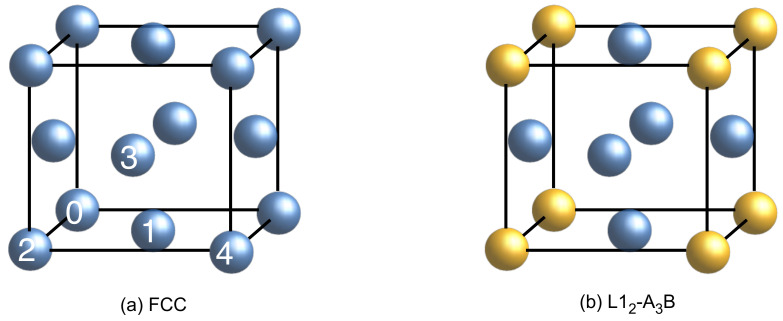
First four coordination shell numbers in fcc lattice (**a**) and the atomic schematics of the L1_2_-A_3_B intermetallic compound (**b**). The Arabic numerals 1, 2, 3, and 4 on the sphere of (**a**) present the coordination shell indexes 110, 200, 211, and 220, respectively, while “0” is the reference site. The positions of B atoms are indicated with golden spheres, while the positions of A atoms are indicated with blue spheres in (**b**).

In order to investigate the solute pair interactions in fcc single-matrix FeCoNiAl_1−*x*_Ti_*x*_ HEAs, the atomic interactions of solute pairs in the fcc Ni matrix were taken into account and calculated using supercell models. The first four nearest neighboring atoms (labeled by Arabic numerals 1, 2, 3, and 4) from a reference site (labeled by Arabic numeral 0) are shown in a conventional fcc cell in Figure 1a, which has been proved to be sufficient for reproducing the atomic environment of FeCoNiAl_1−*x*_Ti_*x*_ HEAs [46]. The atomic configuration of L1_2_-A_3_B is also shown in Figure 1b, where one can see the ordering at the second and fourth coordination shells.

Using Equations (1) and (2), the atomic interactions for all solute pairs were calculated and listed in Table 1. One can see there exists positive atomic interaction energies for all nearest neighboring solute pairs, meaning that the nearest neighboring solute pairs were repulsive in the fcc Ni matrix. These repulsive interactions would contribute to the atomic ordering as well as the formation of precipitates [27,47,48]. At the time time, it is worth noting that the Co–Co first nearest neighboring atomic interaction was not as large as other solute pairs, which indicates that the Co atoms should have no ordering tendency in the FeCoNiAl_1−*x*_Ti_*x*_ HEAs. Moreover, it is known that the atomic radii of Al, Co, Fe, Ni, and Ti are 1.25 Å, 1.35 Å, 1.40 Å, 1.35 Å, and 1.40 Å [49], respectively. Among the aforementioned species, Co and Ni have the similar atomic radius, which may be the reason why Co–Co solute pairs showed weak atomic interactions not only at the first nearest neighboring but also at the second, third, and fourth nearest neighboring. As expected, there was a clear correlation between the sizes of the atoms and the local lattice strains, which would affect the atomic interactions and the atomic binding of the atoms.

Moreover, to understand the effect of atom size mismatch on the interactions between atoms in FeCoNiAl_1−*x*_Ti_*x*_ HEAs, we also show the strain-induced interactions obtained from Equation (3) in Table 1. One notices that the strain-induced interactions of Al–X were quite large compared to those of other solute pairs due to the smallest atomic size of Al (1.25 Å) among the componential species in FeCoNiAl_1−*x*_Ti_*x*_ HEAs. This also holds true for Co–Co strain-induced interactions: the Co–Co solute pair interactions were negligible for both total atomic interactions and strain-induced interactions due to the similar atomic size between Co and Ni atoms. Moreover, it is not surprising that the strong strain-induced interactions of Al–X in the Ni matrix due to atomic radius differences between Al and other atoms (Co, Fe, and Ti) played an important role in the atomic ordering in the FeCoNiAl_1−*x*_Ti_*x*_ HEAs. With the local lattice relaxation as obtained in first-principles calculations of atomic interactions, the atomic configurations and order–disorder transitions in FeCoNiAl_1−*x*_Ti_*x*_ HEAs were investigated using Monte Carlo simulations, which is shown in later sections.

### 3.2. The Order–Disorder Transition in Equal-Atomic AlCoFeNi HEAs

We now turn to the prediction of finite-temperature atomic configurations of AlCoFeNi HEAs using Monte Carlo simulations. The Monte Carlo simulations were performed for a box containing 62,500 atoms ([25 × 25 × 25] × 4 conventional unit cell) with periodic boundary conditions from 2300 K down to 300 K, including the first four nearest neighboring pair interactions as listed in Table 1. At each temperature, the system was first equilibrated for 4000 Monte Carlo steps/atom. After that, the statistical data were obtained by averaging over additional 4000 Monte Carlo steps/atom [27].

**Figure 2:**
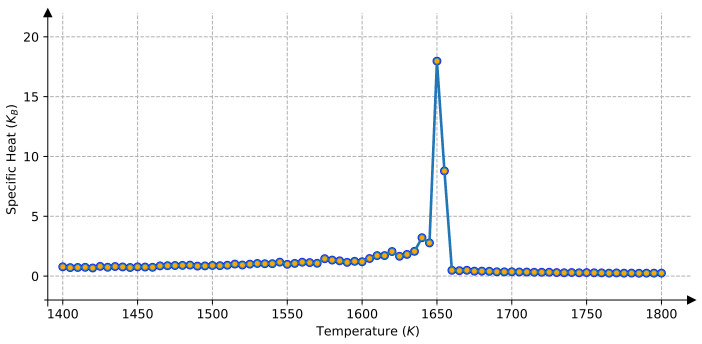
Specific heat capacity of AlCoFeNi HEAs.

With the DFT calculated pair interactions, the heat capacity of the AlCoFeNi HEAs was obtained using Monte Carlo calculations. The configurational contribution to the specific heat, evaluated via the energy fluctuations, is presented in Figure 2. It is obvious that there exists a peak value for specific heat at 1650 K, indicating that there should be a configurational energy change at this temperature. A phase separation at this temperature in AlCoFeNi HEAs can be observed in AlCoFeNi HEAs, and the evolution of the L1_2_ precipitate was also confirmed in the study of Al_*x*_Co_1.5_CrFeNi_1.5_Ti_*y*_ HEAs [50]. However, the mechanism of such a phase evolution has not been discussed in detail. Therefore, the atomic configuration in AlCoFeNiTi HEAs will be shown later in terms of the atom arrangement and short-range order (SRO) parameter $a_{lmn}^{ij}$ of solute pairs, which provides information about the arrangement of atoms in the immediate neighborhood of particular atoms of the lattice [27,46].

### 3.3. The Microstructure of AlCoFeNi at Elevated and Ambient Temperatures

**Figure 3:**
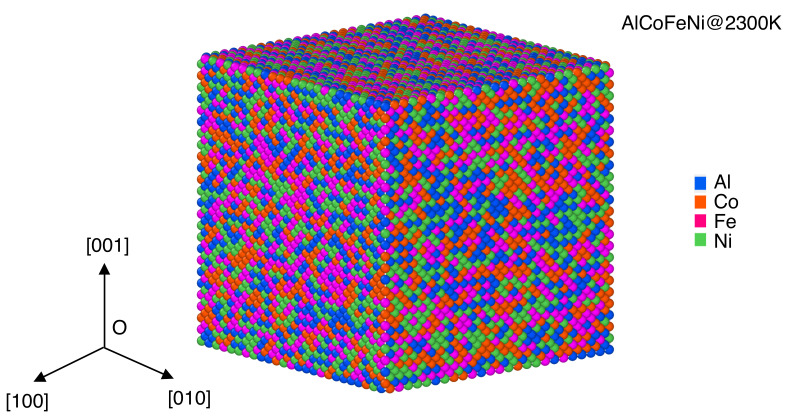
Atomic configuration of AlCoFeNi at 2300 K. Al, Co, Fe, and Ni atoms are indicated with blue, orange, purple, and green spheres, respectively.

In Figure 3, we illustrate the atomic configuration of AlCoFeNi HEAs at an elevated temperature of 2300 K, which is 650 K higher than the order–disorder temperature of AlCoFeNi HEAs. One can see that the elementary elements of AlCoFeNi HEAs are randomly distributed in the cubic box, indicating a disordered atomic configuration in the AlCoFeNi HEAs at the elevated temperature above the order–disorder transition point.

**Figure 4:**
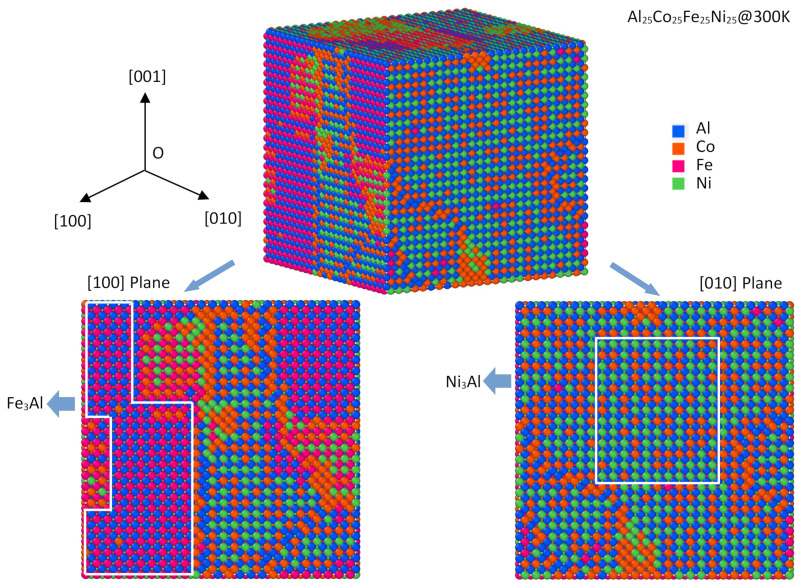
Atomic configuration of AlCoFeNi HEAs at 300 K. The [100] and [010] crystal planes of the three-dimension AlCoFeNi HEAs are also shown at the bottom. Here Al, Co, Fe, and Ni atoms are indicated with blue, orange, purple, and green spheres, respectively. The L1_2_-type precipitates are also framed in the snapshot.

In Figure 4, the atomic configuration of AlCoFeNi HEA at 300 K is illustrated with spheres of different colors, where the L1_2_ ordered structure can be seen in the snapshot. We also depict the [100] and [010] crystal planes of three-dimension AlCoFeNi HEAs at the bottom of the figure. Al, Co, Fe, and Ni atoms are depicted here with blue, orange, purple, and green spheres, respectively. It is interesting that two types of L1_2_ precipitates can be observed in this snapshot at 300 K, i.e., Ni_3_Al and Fe_3_Al. However, one can also see that some of the A and B sites are occupied by solute except Ni, Al, and Fe atoms, for example, one can see that Co atoms replace Al in Ni_3_Al precipitates and Al sites in Fe_3_Al precipitates in the snapshot of Figure 3. Such site preference behaviors of solute in intermetallic compounds transcend the framework of the current paper, it will be a subject of ongoing work [32] and future publications. Moreover, it is obvious that few Co atoms were soluble in the precipitates, however, most Co atoms tended to accumulate and segregate at the interfaces of the precipitates. This is due to the weak atomic interactions between Co atoms, as illustrated in Table 1.

We can therefore conclude that the AlCoFeNi HEAs retain an fcc solid solution down to low temperatures, but they are short-range. According to the Monte Carlo simulation results, the sizes of the aforementioned precipitates are not greater than 35 nm, while the experimental finding indicates that the L1_2_ nano-particles with an average size of 30 nm are embedded in the fcc matrix of Fe–Co–Ni–Al–Ti-based HEA [9]. Our simulated results of precipitate size are in good agreement with the experimental observations.

**Figure 5:**
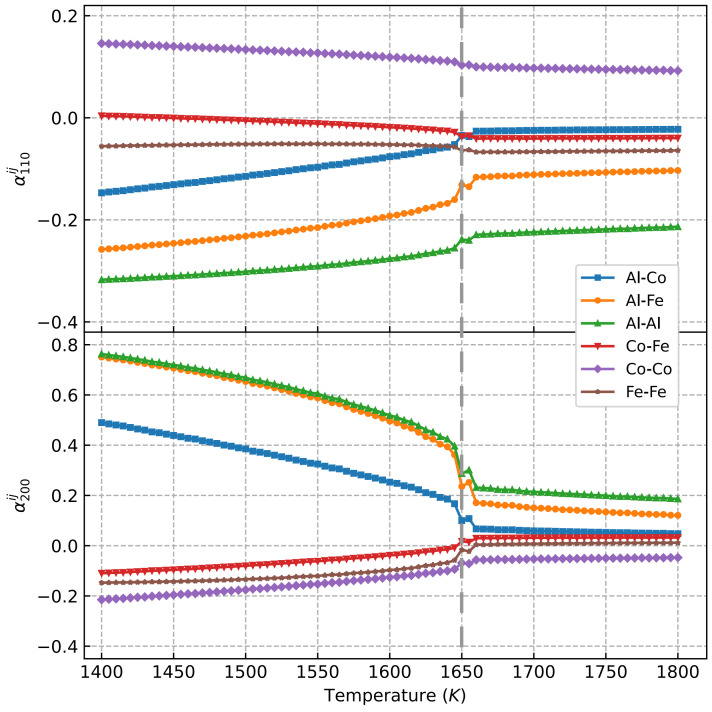
Atomic SRO parameters $a_{lmn}^{ij}$ of AlCoFeNi HEA at the first and second coordination shell from Monte Carlo simulations with the solute interactions from DFT calculations. The dashed line shows the order–disorder transition temperature.

Figure 5 shows the atomic short-range order (SRO) parameters $a_{lmn}^{ij}$ [27] obtained from the Monte Carlo simulations. It clearly indicates that curves of $a_{110}^{\mathrm{Co}-\mathrm{Co}}$, $a_{110}^{\mathrm{Co}-\mathrm{Fe}}$, and $a_{110}^{\mathrm{Fe}-\mathrm{Fe}}$ were very similar, with only small quantitative differences in the whole temperature range. However, $a_{110}^{\mathrm{Co}-\mathrm{Co}}$ was larger and more pronounced than $a_{110}^{\mathrm{Co}-\mathrm{Fe}}$ and $a_{110}^{\mathrm{Fe}-\mathrm{Fe}}$. At the same time, one notices from Table 1 that the Co–Co and Co–Fe pair interactions were negligible (the strongest interaction energy at the first four coordination shells was found to be less than 0.03 eV), which means that the negligible change in SRO parameters was determined by the weak atomic interactions of the solutes. This result agrees with previous findings in [51], where the authors found that the Co–Ni interactions were below 0.01 eV. In the case of Al–Co, Al–Fe, and Al–Al solute pairs, $a_{110}^{\mathrm{Al}-\mathrm{Co}}$, $a_{110}^{\mathrm{Al}-\mathrm{Fe}}$, and $a_{110}^{\mathrm{Al}-\mathrm{Al}}$ decreased at 1650 K, which is the order–disorder transition temperature of AlFeCoNi HEA.

If we look at the SRO parameter in the second coordination shell, which governs the L1${}_{2}$ ordering in the fcc matrix (the nearest A–A sites in L1_2_ A_3_B are located at the second nearest neighboring of the fcc lattice), one can see the dramatic changes in $a_{200}^{\mathrm{Al}-\mathrm{Al}}$ and $a_{200}^{\mathrm{Al}-\mathrm{Fe}}$ at the order–disorder transition temperature, i.e., 1650 K. Clearly, the ordering of Al–Al and Al–Fe solute pairs in the second coordination shell was needed for the formation of L1_2_ Ni_3_Al and Fe_3_Al. The thermodynamic stability of some L1_2_ Ni_3_X intermetallic compounds have been investigated previously [32] and a more comprehensive investigation on the stability of L1_2_-A_3_B intermetallic compounds is shown in later sections.

### 3.4. Alloying Effect of Ti on The Microstructure Evolution of FeCoNiAl_1−x_Ti_x_ HEAs

**Figure 6:**
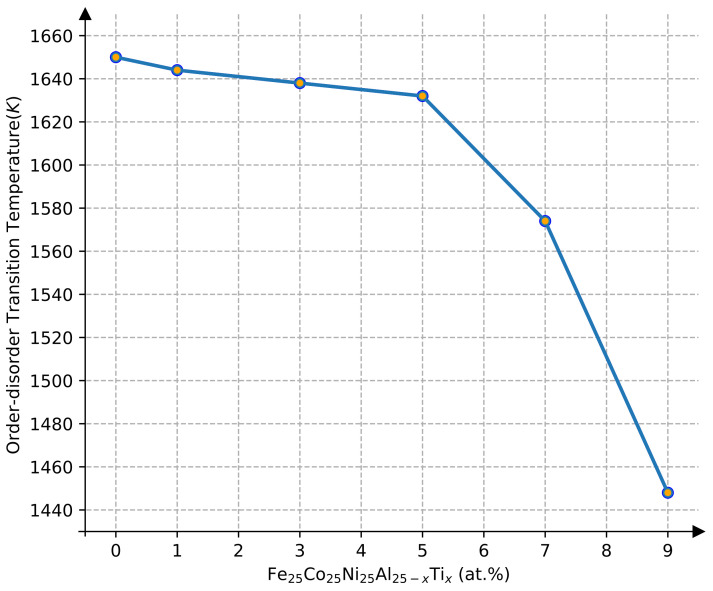
Order–disorder transition temperature as a function of Ti contents in FeCoNiAl_25−*x*_Ti_*x*_ HEAs.

The effect of Ti content on the transition temperature was investigated in the range of 1–9 Ti at.% using Monte Carlo simulations, while the contents of Ti and Al were kept balanced in the FeCoNiAl_1−*x*_Ti_*x*_ HEAs. In the following discussion, the FeCoNiAl _1−*x*_Ti_*x*_ was also written as Fe_25_Co_25_Ni_25_Al_25−*x*_Ti_*x*_ for quasi-quaternary-type equiatomic HEAs. The concentration dependence of the order–disorder phase transition temperature is shown in Figure 6. It was found that the order–disorder transition temperature decreased smoothly with the concentration of Ti in the Fe_25_Co_25_Ni_25_Al_25−*x*_Ti_*x*_ HEAs in the range of 0–5 Ti at.%, while it declined dramatically with the concentration of Ti when the Ti content was higher than 5 Ti at.%. One should note that L1_2_Ni_3_Ti (−0.429 eV) has significantly lower formation enthalpy compared to the Ni_3_Al (−0.475 eV), indicating that Ni_3_Ti is much more stable than Ni_3_Al [32]. This means that the L1_2_ of the Fe_25_Co_25_Ni_25_Al_25−*x*_Ti_*x*_ HEAs can be stabilized by adding or increasing Ti. The calculated results are subsequently in agreement with the experimental observations, in which the as-heat-treated Al_01_Ti_04_ and Al_02_Ti_03_ alloys exhibited stable L1_2_ precipitates [5].

**Figure 7:**
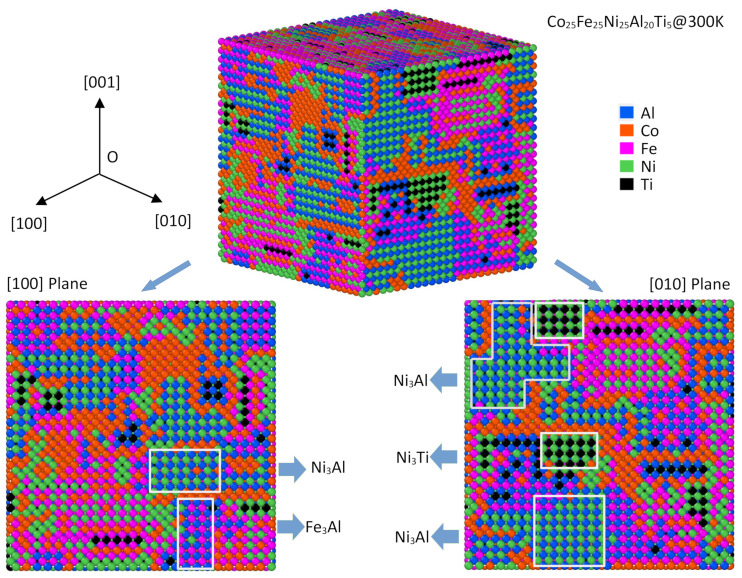
Atomic configuration of AlCoFeNiTi HEAs at 300 K. The [100] and [010] crystal planes of the three-dimension AlCoFeNiTi HEAs are also shown at the bottom. Here, Al, Co, Fe, Ni, and Ti atoms are indicated with blue, orange, purple, green, and black spheres, respectively. The L1_2_-type precipitates are also framed in the snapshot.

The snapshots of the Monte Carlo simulation box of Fe_25_Co_25_Ni_25_Al_20_Ti_5_ HEA at 300 K are shown in Figure 7. One notices that there was a phase separation transition where the L1_2_ ordered structure precipitated in Fe_25_Co_25_Ni_25_Al_20_Ti_5_ HEA. The main chemical compositions of the ordered structure were indeed Ni_3_Al, Ni_3_Ti, and Fe_3_Al precipitates, as framed in Figure 7. Moreover, it is the same as AlCoFeNi HEAs, one can also see the accumulation and segregation of Co atoms at the interfaces of the precipitates in Fe_25_Co_25_Ni_25_Al_20_Ti_5_ HEA due to the weak atomic interactions between Co atoms.

**Figure 8:**
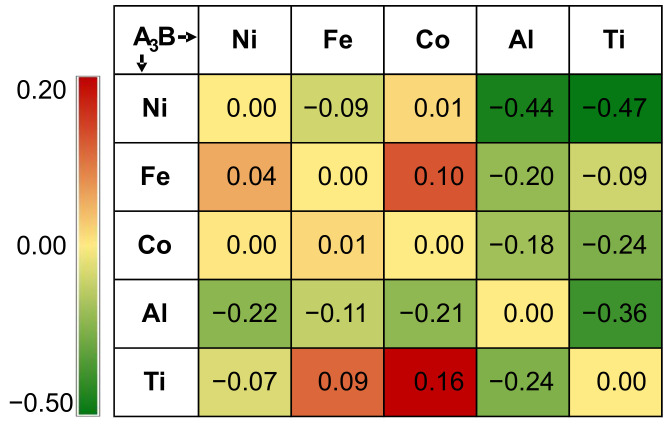
A color map of the formation enthalpies (eV/atom) of L1_2_-A_3_B precipitates in AlCoFeNiTi HEAs. The color scale ranges from green to red, with dark green indicating a formation energy value of −0.50 eV and dark red a formation energy value of 0.20 eV.

The atomic interactions of any two of these components are shown in Table 1, which indicates that the atomic interactions govern the ordering evolution and play a critical role in the formation of L1_2_-type precipitates. To be more quantitative, the thermodynamic properties of L1_2_ intermetallic compounds formed by any two of the components in AlFeCoNiTi HEAs are also calculated and shown in Figure 8 as a form of color map. It may be seen from the formation energy color map that the formation enthalpies of Ni_3_Al and Ni_3_Ti were found to be −0.44 eV and −0.47 eV, respectively. Both of the formation enthalpies were the most stable chemical compositions in L1_2_ in AlFeCoNiTi HEAs, which is in agreement with the snapshots of the atomic results in Figure 4 and Figure 7, where L1_2_ Ni_3_Al and Ni_3_Ti are confirmed. The formation enthalpy of Fe_3_Al was −0.20 eV, which was found in the AlCoFeNi HEA at low temperature. Moreover, one can find that the intermetallic compounds containing Al and Ti have lower formation enthalpies, which means that they should be stable at low temperatures in AlCoFeNiTi HEAs.

In [32], it is reported that Ni_3_Al could be the most stable chemical composition in the L1_2_–Ni_3_X-type intermetallic compounds selected in the list (X = Al, Ti, Mo, Cr, Mo, Fe). Specifically, the formation energy of L1_2_–Ni_3_Al at 0 K was −0.43 eV/atom, which indicates a strong formation tendency of the L1_2_ structure in Ni–Al alloys as well as other alloys containing Ni and Al. In this study, it was also found that the L1_2_–Ni_3_Al precipitates formed at 300 K in AlCoFeNi HEAs, as shown in the [010] crystal plane of Figure 4. However, the sub-lattices of Ni and Al in L1_2_–Ni_3_Al precipitates can also be replaced by other elements, for instance, Fe, Co, or Ti, which are also discussed in [32]. In [52], the formation energies of Fe–Al intermetallic compounds are investigated using first-principles calculations and CALPHAD simulations. The formation energy of L1_2_–Fe_3_Al at 0 K was found to be −0.19 eV/atom, before dropping down to −0.20 eV/atom at 298 K. This means that the L1_2_ structure exhibits a high stability in Fe–Al alloys as well as other alloys containing Fe and Al. Moreover, L1_2_–Fe_3_Al had a stronger formation tendency at the higher temperature (298 K).

**Figure 9:**
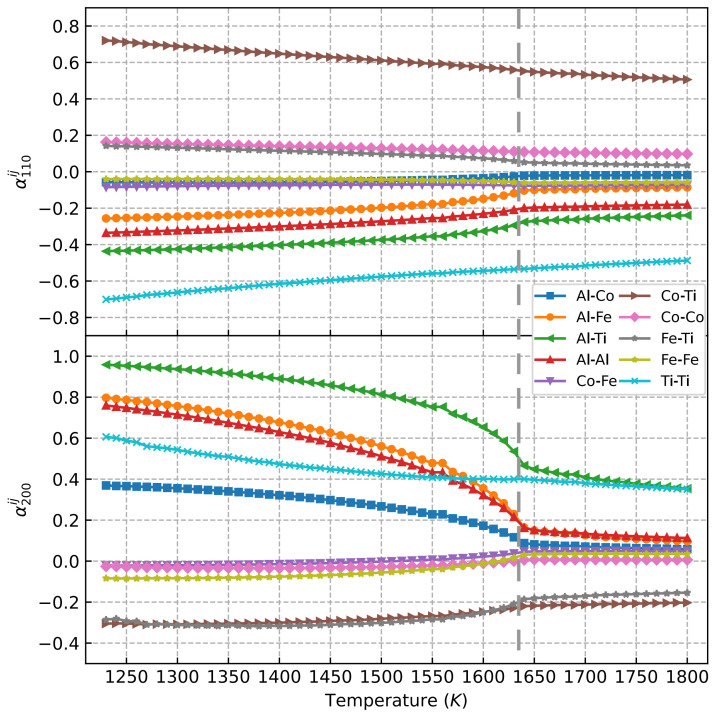
Atomic SRO parameters $a_{lmn}^{ij}$ of Co_25_Fe_25_Ni_25_Al_20_Ti_5_ HEA at the first and second coordination shells from Monte Carlo simulations with the solute interactions from DFT calculations. The dashed line shows the order–disorder transition temperature.

One can still check the SRO parameters again in AlFeCoNiTi HEAs, as they are shown in Figure 9. The SRO parameters for the first coordination shell are similar above and below the transition temperature, i.e., 1632 K. The dramatic change in Al–Al, Al–Ti, Al–Fe, and Al–Co at the second coordination shell can be seen, indicating an atomic ordering for Al, Ti, Fe, and Co in AlFeCoNiTi HEAs, as shown in Figure 7, where Ni_3_Al, Ni_3_Ti, and small volumes of Al_3_Fe and Fe_3_Co were found to be stable at ambient temperature.

### 3.5. The Verification from CALPHAD

**Figure 10:**
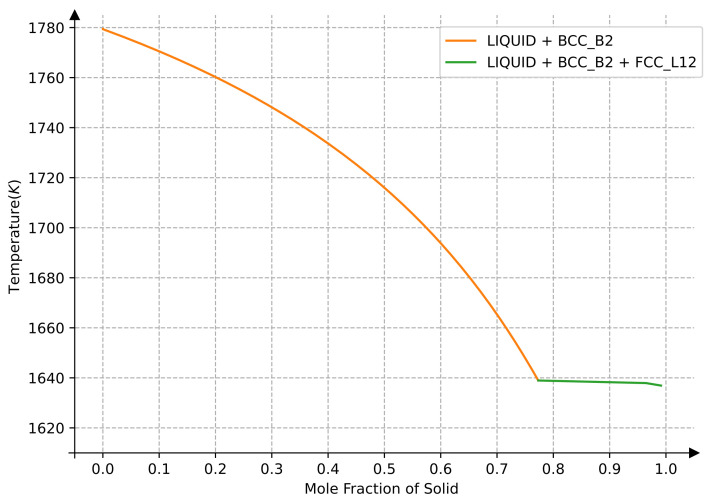
The solidification of an equal-atomic AlCoFeNi alloy.

As shown in Figure 10, the Sheil solidification curve was calculated to predict the L1_2_ precipitation temperature of alloy AlCoFeNi. Our calculated results indicate that the phase precipitation sequence of this alloy is Liquid–Bcc–L1_2_, while the predicted precipitation temperature of L1_2_ is 1640 K. This result is in good agreement with the temperature (1650 K) calculated from the Monte Carlo simulation. Due to the limitation of current Monte Carlo simulations of fcc-based phase transformation, some disagreements between the CALPHAD simulated results based on the current databases and Monte Carlo as well as experimental results are still present [5].

**Figure 11:**
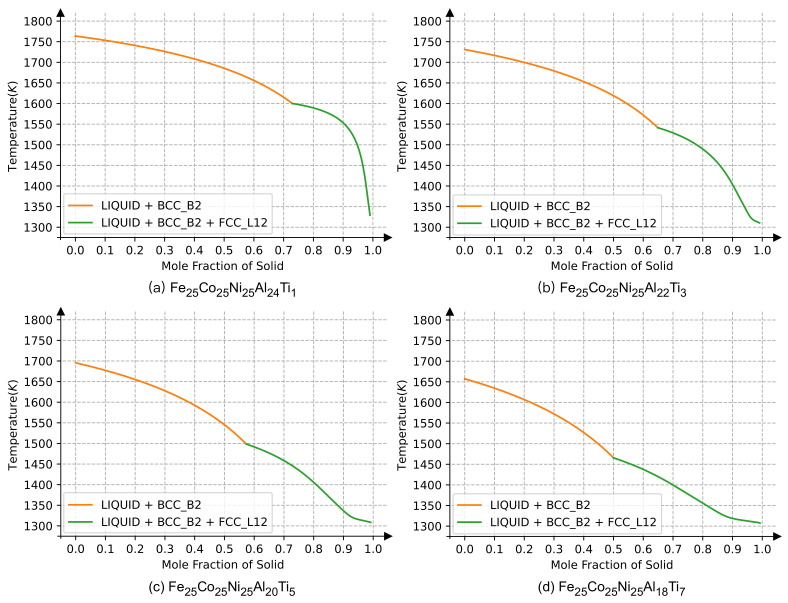
The solidification of the equal-atomic Co_25_Fe_25_Ni_25_Al_25−*x*_Ti_*x*_ alloy. Here, Ti is substituted for the content of a quarter Al in Co_25_Fe_25_Ni_25_Al_25−*x*_Ti_*x*_.

Figure 11 shows the alloying effect of the Ti element on the precipitation temperature of the L1_2_ phase. Four alloys with different compositions were obtained by replacing Al with Ti: Co_25_Fe_25_Ni_25_Al_24_Ti_1_, Co_25_Fe_25_Ni_25_Al_22_Ti_3_, Co_25_Fe_25_Ni_25_Al_20_Ti_5_, and Co_25_Fe_25_Ni_25_Al_18_Ti_7_. The calculated precipitation temperatures of the L1_2_ phase were 1600 K, 1540 K, 1490 K, and 1465 K, respectively. The precipitation temperature decreased with increasing contents of Ti. The order–disorder transition temperatures for the four aforementioned HEAs in the Monte Carlo simulation were 1644 K, 1638 K, 1632 K, and 1574 K, respectively. It is worth noting that the results from the CALPHAD method were much higher than the results calculated using the Monte Carlo simulation, as shown in Figure 6, when the Ti content was less than 5%. One reason could be that the severe lattice distortion caused by Ti alloying significantly changed the atomic configuration, which is a sensitive parameter for Monte Carlo simulations in comparison to CALPHAD calculations.

Another interesting finding from CALPHAD modeling was that the Laves phase began to precipitate at about 1300 K when the Ti content was large enough. Laves are phases with high hardness but poor toughness, they could thus form a disadvantageous precipitate for structural applications. Therefore, the modulation of the Ti content would be an effective way to affect the microstructure and mechanical properties of the HEAs. However, although an investigation into the higher Ti content in Co_25_Fe_25_Ni_25_Al_25−*x*_Ti_*x*_ transcends the scope of the present paper, it would be an interesting subject for future investigations.

## 4. Conclusions

The atomic interactions and order-disorder transition in the FeCoNiAl_25−*x*_Ti_*x*_ HEAs have been investigated by using first-principles calculations and thermodynamic simulations comprehensively. The following conclusions may be drawn:

(1) The chemical ordering in fcc-type FeCoNiAl_1−*x*_Ti_*x*_ HEAs is short-range, while strain-induced interactions play an important role in the atomic ordering. Co atoms were found to accumulate at the interfaces between precipitates and matrix due to their weak interaction in FeCoNiAl_1−*x*_Ti_*x*_, the strong interactions of Al–Al and Ti–Ti atom pairs caused Al and Ti to act as the main components for forming the L1_2_ precipitates.
(2) The presence of the L1_2_ phase and the chemical composition of L1_2_ have been investigated comprehensively for the FeCoNiAl_1−*x*_Ti_*x*_ high-entropy alloys. The site occupation in the L1_2_ phase was found to be complex, and it requires further study. The order–disorder transition temperature from first-principles-based Monte Carlo simulations was 1650 K in FeCoNiAl HEAs, which is also consistent with the CALPHAD results. Moreover, the transition temperature decreased with increasing Ti contents in FeCoNiAl_1−*x*_Ti_*x*_ HEAs.
(3) The order–disorder transition results are in line with existing experimental observations, which means that a selected computer-aided design of the chemical composition can be used for further optimization of the microstructure characteristics in the FeCoNiAl_1−*x*_Ti_*x*_ HEAs.

However, the order–disorder transition in FeCoNiAl_1−*x*_Ti_*x*_ HEAs is complicated. Beyond what was performed in the current investigation, it also involves element site occupation, element segregation at interfaces, and phase competition. All of these require systematic study in further research.

## Figures and Tables

**Table 1:** Pair interaction (in eV) of solute pairs in fcc Ni matrix. lmn: Coordination shell index.

	$V_{p}^{\mathrm{total}}$				$V_{p}^{\mathrm{SI}}$			
lmn	110	200	211	220	110	200	211	220
Al–Al	0.243	−0.009	0.003	−0.013	−0.099	−0.002	−0.003	0.044
Al–Co	0.063	−0.010	−0.016	−0.012	−0.083	−0.003	0.000	−0.012
Al–Fe	0.077	−0.021	−0.013	0.006	−0.173	−0.003	0.002	0.044
Al–Ti	0.127	−0.024	−0.012	0.007	−0.144	0.003	−0.195	−0.001
Co–Co	0.003	−0.002	0.001	0.010	0.000	−0.001	0.001	−0.001
Co–Fe	0.029	0.001	0.004	−0.002	−0.018	0.001	−0.001	0.001
Co–Ti	0.203	−0.082	−0.022	−0.020	−0.011	−0.005	−0.007	−0.012
Fe–Fe	0.115	−0.015	0.015	0.010	−0.014	0.000	0.001	0.000
Fe–Ti	0.129	−0.073	−0.018	−0.013	−0.025	−0.004	−0.003	−0.016
Ti–Ti	0.367	−0.129	−0.052	−0.054	0.087	−0.053	−0.059	0.087

## Data Availability

Not applicable.

## Abbreviations

The following abbreviations are used in this manuscript:

HEAs	High-entropy alloys
CALPHAD	Calculation of phase diagrams
VASP	Vienna ab initio simulation package
PAW	Projector augmented-wave
GGA	Gerneralized gradient approximation
PBE	Perdew–Burke–Ernzerhof
DFT	Density functional theory
MC	Monte Carlo
SRO	Short-range order
BCC	Body-centered cubic
FCC	Face-centered cubic
HCP	Hexagonal close-packed
